# Retraction Notice to: circFLT1 and lncCCPG1 Sponges miR-93 to Regulate the Proliferation and Differentiation of Adipocytes by Promoting lncSLC30A9 Expression

**DOI:** 10.1016/j.omtn.2026.102877

**Published:** 2026-03-04

**Authors:** Zihong Kang, Sihuang Zhang, Enhui Jiang, Xinyu Wang, Zhen Wang, Hong Chen, Xianyong Lan

## Main text

(Molecular Therapy: Nucleic Acids *22*, 484–499; December 4, 2020)

This article has been retracted at the request of the editor-in-chief. During an investigation of related articles, similarities were found between the figures in this article. Image analysis performed by the editorial office revealed evidence of image duplication involving Figures 2F, 2H, 2K, 3I, 4F, 4H, 4K, 4L, 5E, 5F, 7G, 7H, 8B, S2E, S2G, S2K, S6F, S6H, and S6L of this *Molecular Therapy Nucleic Acids* article. The authors provided available original data upon request; however, the initial concerns could not be alleviated. This reuse of data without appropriate attribution represents a severe abuse of the scientific publishing system.

